# Human Papillomavirus 16 E6 Contributes HIF-1α Induced Warburg Effect by Attenuating the VHL-HIF-1α Interaction

**DOI:** 10.3390/ijms15057974

**Published:** 2014-05-07

**Authors:** Yi Guo, Xiangkai Meng, Jiaming Ma, Yahong Zheng, Qian Wang, Yanan Wang, Hong Shang

**Affiliations:** 1 Department of Gynecology, First Affiliated Hospital of China Medical University, Shenyang 110001, China; E-Mails: xiangkaimeng@hotmail.com (X.M.); Yahong516@163.com (Y.Z.); 2 Department of Pain Management, Shengjing Hospital of China Medical University, Shenyang 110022, China; E-Mail: mming517@126.com; 3 Key Laboratory of AIDS Immunology of Ministry of Health, Department of Laboratory Medicine, First Affiliated Hospital of China Medical University, Shenyang 110001, China; E-Mails: qianwangyj@163.com (Q.W.); wangyn@163.com (Y.W.)

**Keywords:** HPV16 E6, hypoxia inducible factor-1 (HIF-1), Warburg effect, von Hippel-Lindau tumor suppressor gene (VHL)

## Abstract

Cervical cancer is still one of the leading causes of cancer deaths in women worldwide, especially in the developing countries. It is a major metabolic character of cancer cells to consume large quantities of glucose and derive more energy by glycolysis even in the presence of adequate oxygen, which is called Warburg effect that can be exaggerated by hypoxia. The high risk subtype HPV16 early oncoprotein E6 contributes host cell immortalization and transformation through interacting with a number of cellular factors. Hypoxia-inducible factor 1α (HIF-1α), a ubiquitously expressed transcriptional regulator involved in induction of numerous genes associated with angiogenesis and tumor growth, is highly increased by HPV E6. HIF-1α is a best-known target of the von Hippel-Lindau tumor suppressor (VHL) as an E3 ligase for degradation. In the present work, we found that HPV16 E6 promotes hypoxia induced Warburg effect through hindering the association of HIF-1α and VHL. This disassociation attenuates VHL-mediated HIF-1α ubiquitination and causes HIF-1α accumulation. These results suggest that oncoprotein E6 plays a major role in the regulation of Warburg effect and can be a valuable therapeutic target for HPV-related cancer.

## 1. Introduction

It is well known that cancer cells have different metabolism patterns compared to their normal counterparts. Cancer cells consume more glucose to obtain energy by glycolysis even in the present of proper oxygen, this unique metabolic phenotype is referred to as the Warburg effect [1]. This reprogramming of metabolic pathways in tumor cells ensures sufficient bioenergy, a favorite redox state providing proteins, nucleic acids, lipids, and other macromolecules. Whereas the mechanisms of the Warburg effect are still not fully understood, the specialized tumor hypoxic microenvironment may represent a well-recognized mechanism [2]. The cellular response to hypoxia is mediated by a transcriptional complex referred to as hypoxia inducible factor 1 (HIF-1), which consists of HIF-1α (and subsequently HIF-2α and HIF-3α) as the O2-responsive subunit and HIF-1β subunits as the constitutively expressed subunit [3]. Under normal physiological conditions HIF-1α is the best-known degradation target of the von Hippel-Lindau tumor suppressor gene (VHL)-containing E3 ligase [4]. At normal oxygen level, HIF-1α is hydroxylated at the proline residues by proline hydroxylase domain (PHD) proteins [5]. The prolyl-hydroxylated HIF-1α is recognized by VHL, leading to poly-ubiquitination and degradation. Under hypoxic conditions, however, PHD activity is inhibited, and HIF-1α then accumulates and dimerizes with HIF-1β, forms the active HIF-1 complex and translocates to the nucleus where the dimmer functions as a transcription factor. Its best-known target genes encode proteins involved in glycolysis (e.g., phosphoglycerate kinase), glucose transport (Glut-1), angiogenesis (vascular endothelial growth factor (VEGF)) and erythropoiesis (erythropoietin); that is, proteins that mediate the cellular response and adaptation to hypoxic conditions [6]. In addition to this hypoxia-dependent stabilization system, a range of other genetic alterations, such as activation of oncogenes, loss of function of tumor suppressor genes, and mutations in metabolic enzyme genes can also activate HIF-1α and thereafter promote the Warburg effect. Activation of HIF-1α contributes to the Warburg effect through coordinated upregulation of glycolysis and downregulation of oxidative phosphorylation.

The high risk type of human papillomaviruses (HPVs) are etiological agents most ano-genital cancers and for a subset of head and neck neoplasias [7,8]. The activity of HPV16 early proteins E6 and E7 plays the crucial role for host cell immortalization and transformation by inactivating the tumor suppressors, p53 and pRB, respectively [9]. Persisting expression of these two oncoproetins is essential for HPV related cancers throughout the development. Furthermore, inhibition of E6/E7 expression impedes the growth of HPV positive cancer cells [10]. Particularly, E6 not only recruits a ubiquitin protein ligase E6AP, and the resulted complex targets the p53 tumor suppressor protein for proteasome mediated degradation [11,12], but also impedes p53 acetylation by hindering the interaction of p53 and ING4 [13].

Although it has been demonstrated that the level of HIF-1α protein is increased under hypoxia condition when HPV oncogenes are present [14], the deregulation of cellular metabolism in HPV mediated cervical carcinoma is still elusive to us. Here we reported that HPV16 E6 contributes the Warburg effect via disturbing the interaction of HIF-1α and VHL.

## 2. Results and Discussion

### 2.1. HPV16 E6 Forms Complex with HIF-1α and Enhances Its Expression

**Figure 1.**
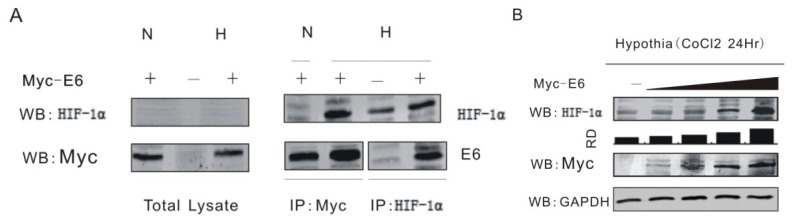
HPV16 E6 forms complex with HIF-1α and enhances its expression. (**A**) HPV16 E6 coimmunoprecipitates with HIF-1α results showed that E6 associates with endogenous HIF-1α. Transfected HEK 293T cells cultured under normoxia (N) or hypoxia (H) condition were immunoprecipitated (IP) using anti-myc (9E10) or anti-HIF-1α monoclonal antibody and subsequently Western blotted (WB) with anti-HIF-1α or anti-myc antibodies (right panels). Total cell lysate (1%) was blotted with anti-myc or anti-HIF-1α antibodies (left panels). We performed coimmunoprecipitation using anti-myc or anti-HIF-1α antibody separately. The precipitates were analyzed by Western blotting anti-HIF-1α and anti-myc antibodies; (**B**) The HPV16 E6 regulates HIF-1α expression in a dose-dependent manner. Ten million hypoxia treated HEK 293T cells were transfected with different doses of plasmid Myc-E6 normalized by the Myc vector and maintained under hypoxic condition. After 24 h of transfection, the cells were harvested and lysed for Western blot (WB) analysis using antibodies against HIF-1α, myc, or GAPDH. The data show that HIF-1α expression was increased by E6 in a dose dependent manner. RD, relative density.

It has been showed that HPV especially its oncoprotein E6 enhances the expression of HIF-1α from either clinical specimen or molecular experiments [14,15]. First, we investigated whether HPV16 E6 forms a complex with HIF-1α by using coimmunoprecipitation assays. HEK 293T cells were transfected with or without expression plasmid encoding myc-tagged E6 during normoxia (N) or 100 μM cobalt chloride-induced hypoxia (H) condition. Forty-eight hours posttransfection, we performed coimmunoprecipitation using anti-myc or anti-HIF-1α antibody separately. The result showed that endogenous HIF-1α associates with HPV16 E6 in cells (Figure 1A). We also detected the effect of the transient expression of HPV16 E6 on endogenous HIF-1α level in HEK 293T cell lines. Consistent with the previous report, the transient expression of HPV16 E6 increased endogenous levels of HIF-1α in a dose-dependent manner (Figure 1B).

### 2.2. HPV16 E6 Augments HIF-1α Induced Glycolysis

**Figure 2.**
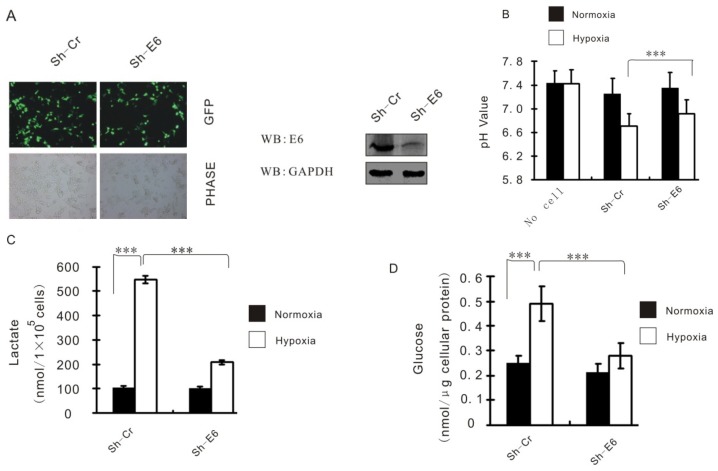
HPV16 E6 augments HIF-1α induced glycolysis. (**A**) HPV16 E6 knockdown and control cells of CaSki by lentivirus-delivered shRNA. GFP expression was determined by fluorescence microscopy; (**B**) CaSki cells expressing either control shRNA (Sh-Cr) or the indicated E6 shRNA (Sh-E6) were cultured under normoxic or hypoxic conditions for 24 h. pH value of the culture medium was then measured. The data are shown as mean ± SD of three independent experiments; (**C**) Caski cells expressing either control shRNA or E6 shRNA were cultured under normoxic or hypoxic conditions for 24 h. Levels of lactate in the culture medium were then measured and normalized to cell number. Data shown are mean ± SD (*n* = 3); (**D**) Caski cells expressing either control shRNA or E6 shRNA were cultured under normoxic or hypoxic conditions for 24 h. Intracellular glucose levels were then measured and normalized based on protein concentration. Data shown are mean ± SD (*n* = 3). *** means *p* < 0.01.

Stable CaSki cell lines carrying HPV16 E6 knockdown (Sh-E6) or control (Sh-Cr) were made by transduction with shRNA-containing lentivirus then selection (Figure 2A). Hypoxia causes increased glycolysis, which is characterized by acidification of the culture medium, increasing of glucose uptake and lactate production, although the cell growth rate remains practically unaltered. However, all the metabolism effects were significantly reversed when HPV16 E6 gene was knockdown in Caski cells (Figure 2B–D), suggesting that HPV16 E6 functions as a major contributor of hypoxia-enhanced glycolysis.

### 2.3. The Effect of HPV16 E6 on Hypoxia-Enhanced Glycolysis Is HIF-1α Dependent

**Figure 3.**
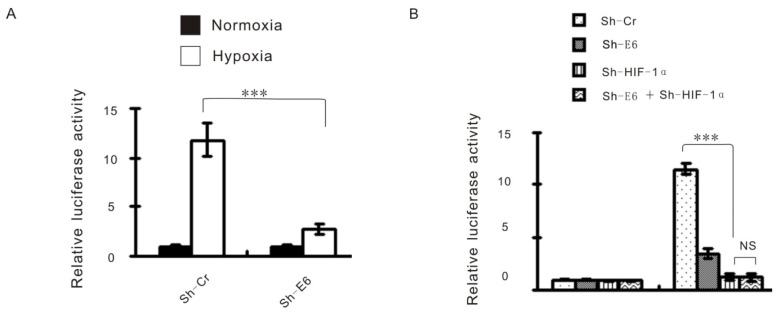

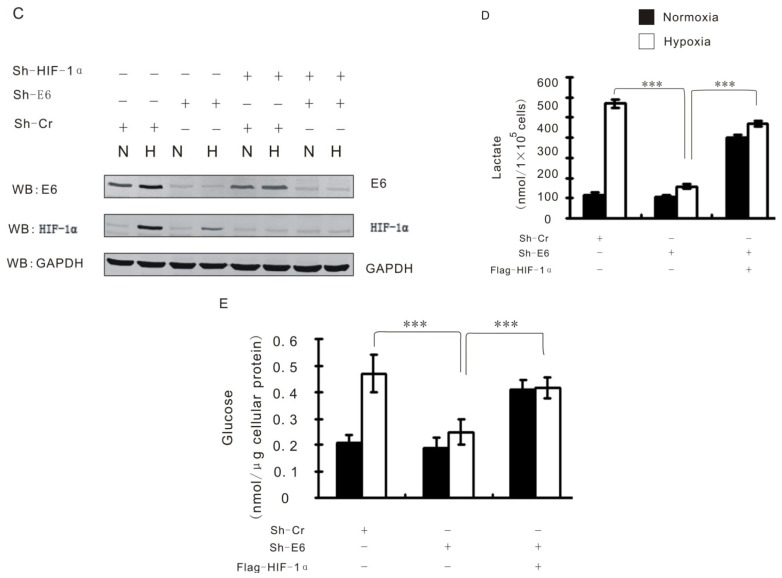
The Effect of HPV16 E6 on Hypoxia-Enhanced Glycolysis is HIF-1α Dependent. (**A**) Caski cells expressing either control shRNA or E6 shRNA were cotransfected with the indicated reporter constructs and Renilla luciferase plasmid. Twelve hours later, cells were cultured under normoxic or hypoxic conditions for an additional 24 h. Reporter activity was measured and plotted after normalizing with respect to Renilla luciferase activity. Shown data are mean ± SD (*n* = 3); (**B**) Caski cells expressing control shRNA, E6 shRNA, HIF-1α shRNA, or both E6 and HIF-1α shRNAs were cotransfected with the HRE reporter construct and Renilla luciferase plasmid. Twelve hours later, cells were cultured under normoxic or hypoxic conditions for an additional 24 h. Reporter activity was measured and plotted after normalizing with respect to Renilla luciferase activity. Shown data are mean ± SD (*n* = 3). The term “NS” indicates no significance; (**C**) Caski cells expressing control shRNA, E6 shRNA, HIF-1α shRNA, or both E6 and HIF-1α shRNAs were cultured under normoxic or hypoxic conditions for 24 h before cell lysates were analyzed by Western blotting; (**D**,**E**) Caski cells expressing either control shRNA or E6 shRNA were transfected with or without Flag-HIF-1α. Twenty-four hours later, cells were cultured under normoxic or hypoxic conditions for an additional 24 h. Lactate levels in the culture medium (**D**) and intracellular glucose levels (**E**) were then measured. Data shown are mean ± SD (*n* = 3). *** means *p* < 0.01.

HIF-1α as a transcriptional factor can function through interaction with specific *cis*-acting DNA binding sites, referred to as HIF-1α response elements (HREs), which are located within the promoter regulatory sequences of its target genes [16,17]. We then use HRE-luciferase reporter system to check the effect of HPV16 E6 on HIF-1α-regulated luciferase expression. Consistent with the expected result, hypoxia strongly increased luciferase expression, this increase was strongly suppressed by HPV16 E6 knockdown (Figure 3A). Moreover, we reduced HIF-1α expression using its specific shRNA, HPV16 E6 knockdown failed to show any effect on reporter activity under hypoxia (Figure 3B), implying that HPV16 E6 indeed affects hypoxia-induced transcription via HIF-1α. Intriguingly, we also showed that hypoxia-induced HIF-1α expression was largely attenuated by HPV16 E6 knockdown (Figure 3C). This result further confirms that HPV16 E6 is essential for hypoxia-induced HIF-1α upregulation. To investigate whether HPV16 E6 regulates hypoxia enhanced glycolysis really through HIF-1α, exogenous HIF-1α was introduced into HPV16 E6 knockdown cells. Ectopic expression of HIF-1α indeed reversed the effect of E6 knockdown on lactate production (Figure 3D) and glucose uptake (Figure 3E). Thus, these data suggest that the role of HPV16 E6 on hypoxia-enhanced glycolysis is dependent on HIF-1α.

### 2.4. The HPV16 E6 Stabilizes HIF-1α through Attenuating VHL-HIF-1α Association

**Figure 4.**
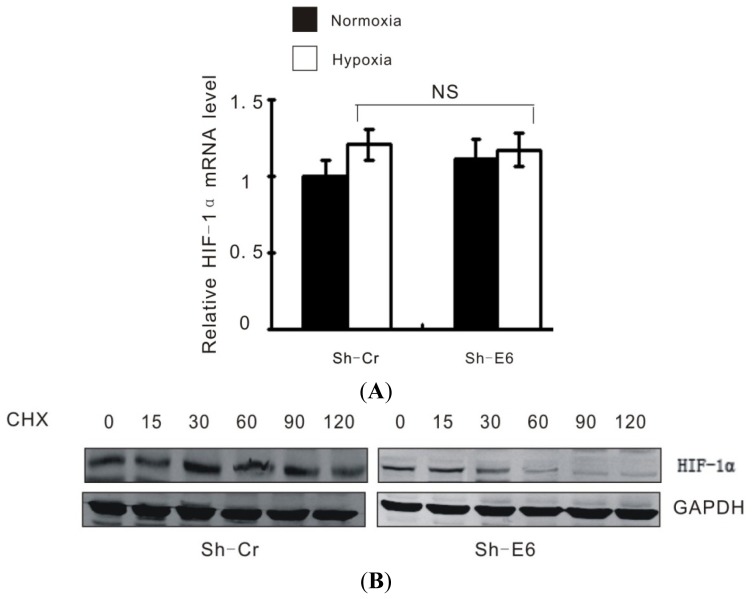

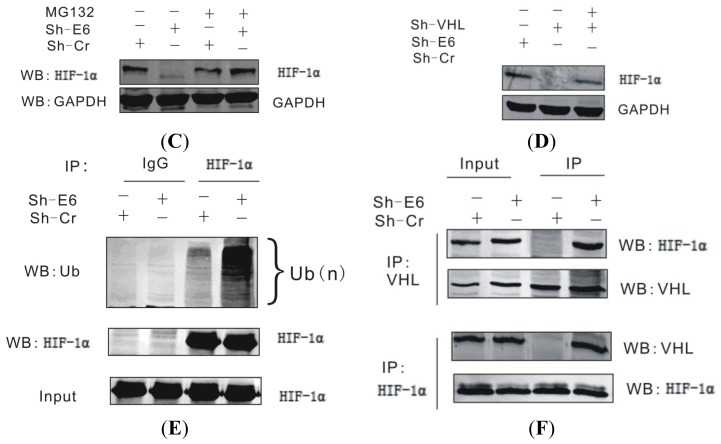
HPV16 E6 stabilizes the HIF-1α by attenuating the VHL-HIF-1α association. (**A**) Caski cells expressing either control shRNA (Sh-Cr) or E6 shRNA (Sh-E6) were cultured under normoxic or hypoxic conditions for 24 h. Total RNA was then subjected to real-time RT-PCR analysis. Data shown are mean ± SD (*n* = 3). The term “NS” indicates no significance; (**B**) Caski cells expressing either control shRNA (Sh-Cr) or E6 shRNA (Sh-E6) were transfected with Flag-HIF-1α. Twelve hours later, cells were cultured under hypoxic conditions for an additional 24 h before they were treated with cycloheximide (20 mg/mL) for the indicated periods of time. Cell lysates were then analyzed by Western blotting; (**C**) Caski cells expressing either control shRNA (Sh-Cr) or E6 shRNA (Sh-E6) were cultured under hypoxic conditions in the presence or absence of MG132 (5 μM) for 24 h. Cell lysates were then analyzed by Western blotting; (**D**) Caski cells expressing control shRNA (Sh-Cr), E6 shRNA (Sh-E6), or both VHL and E6 shRNAs (Sh-VHL + Sh-E6) were cultured under hypoxic conditions for 24 h. Cell lysates were then analyzed by Western blotting; (**E**) Caski cells expressing either control shRNA (Sh-Cr) or E6 shRNA (Sh-E6) were cultured under hypoxic conditions in the presence of MG132 (5 μM) for 24 h. Cell lysates were immunoprecipitated with either control IgG or anti-HIF-1α antibody. The immunoprecipitates and input were analyzed by Western blotting; (**F**) Caski cells expressing either control shRNA (Sh-Cr) or E6 shRNA (Sh-E6) were cultured under hypoxic conditions in the presence of MG132 (5 μM) for 24 h. Cell lysates were immunoprecipitated with anti-VHL, or anti-HIF-1α antibody. The immunoprecipitates and input were analyzed by Western blotting.

It has been shown that HPV E6 enhances activation of HIF-1α under hypoxic conditions [14,15]. However, the mechanism behind HPV16 E6 stabilizing HIF-1α protein expression is still puzzled to us. Experiments indicate that enhanced induction of HIF-1α by HPV is due to increased stability of the protein at post-transcription level [15]. First we checked the HIF-1α mRNA levels of HPV16 E6 knockdown cells and the control cultured both at normoxic and hypoxic condition. Real time PCR analysis did not show differences of HIF-1α mRNA caused by HPV16 E6 knockdown (Figure 4A). However, HPV16 E6 knockdown significantly decreased HIF-1α half-life under hypoxia (Figure 4B). The HPV16 E6 knockdown-induced HIF-1α instability was able to be rescued by the proteasome inhibitor MG132 (Figure 4C), indicating that HPV16 E6 protects HIF-1α from proteasome-dependent degradation. The effect of HPV16 E6 on HIF-1α is VHL dependent, for dual knockdown of HPV16 E6 and VHL recovered HIF-1α degradation under hypoxia (Figure 4D). Furthermore, HPV16 E6 knockdown also greatly increased HIF-1α polyubiquitination under hypoxic conditions (Figure 4E). One mechanism behind this phenomena is that knockdown of HPV16 E6 enhanced the HIF-1α-VHL interactions under hypoxia (Figure 4F). Together, these results indicate that HPV16 E6 attenuates the association of VHL and HIF-1α, thereafter alleviating VHL-mediated HIF-1α ubiquitination and subsequent degradation.

### 2.5. Discussion

The changes of the metabolic pattern named Warburg effect has long been recognized as a hallmark of cancer cells. It has been reported that hypoxia contribute Warburg effect through the regulation of various protein expression. HPV oncogenic protein E6 is an important carcinogenic agent in cervical cancer [4]. HPV E6 promotes cell proliferation through intervening in functions of several cellular agents by protein-protein interactions. Though it has well been established that HPV16 oncoproteins enhance HIF-1α and its down stream gene expression [18]. The detail relationship and mechanism of HPV16 E6 and HIF-1α in contributing to Warburg effect are still need to be elucidated.

In this study, we show that HPV16 E6 plays an essential role in hypoxia-enhanced glycolysis, indicating that HPV16 E6 may be a critical contributor in the regulation of the Warburg effect. Our results reveal that under hypoxic conditions HPV16 E6 forms a complex with HIF-1α and enhances hypoxia-induced HIF-1α expression. Manipulating of the hypoxic response is important for many papillomavirus types, for both high-risk HPV16 and 31, as well as the low risk HPV11 were found to be equally capable of enhancing HIF-1α levels [15]. Regions of hypoxia are known to exist within majority of solid tumors, and the extent of tumor hypoxia correlates with prognosis for a number of tumor types [19,20]. The cellular response to hypoxia involves diverse processes, including glycolysis and angiogenesis, which are closely associated with high metastases and a poor prognosis [21]. HIF-1α level is affected by a wide range of cellular factors and pathways [22,23]. It is has been reported that that HIF-1α can be upregulated by E6 and E7 but only in hypoxia and through protein stabilization using stable cell lines [15]. While others showed that transient transfection of E6 and E7 expression vectors into cervical cancer cell lines increased HIF-1α levels under normoxic conditions but without altering protein stability [18]. It implies that the effect of E6 on HIF-1α protein stabilization is hypoxia-specific that is more likely to reflect conditions *in vivo*.

Metabolism in cancer cells is commonly dysregulated. This metabolic pattern change is brought about through via overlapping yet distinct oncogenic mutation that result in hyperactive oncogenes or inactive tumor suppressors, but it can also be achieved through mutations in metabolic enzymes themselves. Some of metabolic “rewriting” in cancer cells are common to many kinds of tumors, while others appear to be specific to certain tumor types. Here we show that HPV16 E6 contributes Warburg effect by enhancing HIF-1α induced glycolysis. On the contrary, when E6 was transfected in an isogenic ovarian cell resulted in reduced glycolysis, amino acid uptake and fatty acid synthesis [24]. The differences may result from cell type specific or hypoxia specific.

Our experiment shows that HPV16 E6 stabilizes HIF-1α by attenuating its association with VHL, thus avoiding the latter mediated protein degradation. The hypothesis about this mechanism we propose is that there are competing relationships among these three proteins. HPV 16 E6 and VHL can compete to bind HIF-1a. In another word, binding of E6 to HIF-1α will preclude further binding of VHL to HIF-1α. It is worth noting that although the effect of HPV16 E6 on HIF-1α only happens under hypoxia, so as-yet-unidentified hypoxia-responsive factors may also be required for hypoxia-induced HIF-1α expression.

## 3. Experimental Section

### 3.1. Reagents and Antibodies

The following reagents and antibodies are used in the experiments: CoCl_2_, anti-HPV16 E6 (C1P5), anti-HIF-1α and anti-VHL (Santa Cruz, CA, USA), mouse IgG1 (M075-3, MBL), rabbit IgG and anti-Flag (Sigma, St. Louis, MO, USA), anti-GAPDH, anti-Ub (Santa Cruz, CA, USA), MG132 Calbiochem (San Diego, CA, USA), Cycloheximide (Sigma-Aldrich, St. Louis, MO, USA).

### 3.2. Cell Culture and Transfection

CaSki and HEK 293T cells were grown in Dulbecco’s modified Eagle’s medium (DMEM; purchased from Hyclone Logan, Logan, UT, USA) supplemented with 10% fetal bovine serum, 50 U/mL penicillin, 50 μg/mL streptomycin, and 2 mM l-glutamine. The cells were transfected by electroporation with a Bio-Rad Gene Pulser II electroporator (Hercules, CA, USA). To create a hypoxic environment, cells were allowed to grow with 100 μM CoCl_2_ treatment before use.

### 3.3. Immunoprecipitation and Western Blotting

Transfected cells were harvested, washed with ice-cold PBS, and lysed in 0.5 mL ice-cold radioimmunoprecipitation buffer, supplemented with protease inhibitors. Cell debris was removed by centrifugation, lysates were then precleared by end-over-end rotation with normal mouse serum and 30 μL of a 1:1 mixture of protein A-protein G-conjugated Sepharose beads (1 h, 4 °C). Approximately 5% of the lysate was saved for input control. The protein of interest was captured by rotating the remaining lysate with 1 μg of appropriate antibody overnight at 4 °C. Immune complexes were captured with 30 μL of a 1:1 mixture of protein A and protein G Sepharose beads. For Western blot assays, input lysates and immunoprecipitated (IP) complexes were boiled in Laemmli buffer, fractionated by SDS-PAGE, and transferred to a 0.45 μm nitrocellulose membrane. The membranes were then probed with appropriate antibodies followed by incubation with appropriate infrared-tagged secondary antibodies and viewed on an Odyssey imager (LiCor Inc., Lincoln, NE, USA).

### 3.4. Glucose Uptake Assay

Caski cells expressing control shRNA or E6 shRNA were cultured in glucose-free DMEM for 16 h, and then incubated with high-glucose DMEM under normoxic or hypoxic conditions for an additional 24 h. The culture medium was removed, and the intracellular glucose levels were measured using a fluorescence-based glucose assay kit (BioVision, Milpitas, CA, USA) according to manufacturer’s instructions.

### 3.5. Lactate Production Assay

Caski cells expressing control shRNA or E6 shRNA were cultured under normoxic or hypoxic conditions for 24 h. Lactate levels in the culture medium were determined using a fluorescence-based lactate assay kit (BioVision) according to manufacturer’s instructions.

### 3.6. Luciferase Reporter Assay

Twelve million cells were co-transfected by using a Bio-Rad electroporater (Bio-Rad Laboratories, Inc., Hercules, CA, USA) with HRE reporter construct with combinations of different plasmids. At 24 h post-transfection, cells were harvested, washed in PBS, and lysed in cell lysis buffer (BioVision, Inc., Mountain View, CA, USA). Fifty microliter of cell lysate was used for the reporter assay, using an LMaxII384 luminometer (Molecular Devices, Inc., Sunnyvale, CA, USA). The results are shown as the means of the data from three independent experiments.

### 3.7. Lentiviral-Mediated Gene Silencing

For the lentivirus-mediated stable knockdown of HPV16 E6 or HIF-1α, the E6 shRNA sequence (5′-GGACAGAGCCCATTACAATAT-3′) or HIF-1α shRNA sequence (5′-CCGCTGGAGA CACAATCATAT-3′) was inserted into pGIPZ vector respectively according to the manufacture’s instructions (Open Biosystem, Inc., Huntsville, AL, USA), the vector expressing HPV16 E6 or small hairpin RNA was abbreviated as Sh-E6 or Sh-HIF-1α. In addition, a 21-mer oligonucleotide (TCTCGCTTGGGCGAGAGTAAG) that had no significant homology to any known human mRNA in the databases was cloned in the same vector and used as control. Control shRNA is hereinafter abbreviated as sh-C.

Lentiviruses were produced by transient transfection into HEK 293T cells, a total of 2 × 10^6^ HEK 293T cells were seeded in 10-cm-diameter dishes in DMEM (HyClone, Logan, UT, USA) supplemented with 10% FBS and 1% antibiotic-antimycotic and cultured in a 5% CO_2_ incubator for 24 h prior to transfection. A total of 20 μg of plasmid DNA was used for the transfection of each dish, including 1.5 μg of envelope plasmid pCMV-VSV-G (catalog No. 8454; Addgene, Inc., Cambridge, MA, USA), 3 μg of packaging plasmid pRSV-REV (catalog No. 12251 Addgene, Inc., Cambridge, MA, USA), 5 μg of packaging plasmid pMDLg/Prre (catalog No. 12251 Addgene, Inc., Cambridge, MA, USA), and 10.5 μg of lentiviral vector plasmid. The precipitation was formed by adding the plasmids to a final volume of 438 μL of H_2_O and 62 μL of 2 M Cacl_2_, mixing well, adding 500 μL of 2× HEPES-buffered saline, and then incubating at room temperature for 30 min. Chloroquine was added to the 10 mL of plated media with a final concentration of 25 μM at 5 min prior to transfection. The medium was replaced after 12 h with DMEM supplemented with 10% FBS and 10 mM HEPES, and 10 mM sodium butyrate. The medium was replaced again 10 h later using DMEM supplemented with 10% FBS and 10 mM HEPES. The conditioned medium was collected four times at 12 h interval, filtered through 0.45 μm pore-size cellulose acetate filters, and stored on ice. The virus was concentrated by spinning at 70,000× *g* for 2.5 h. The concentrated virus was resuspended in RPMI then used to infect 10^6^ cells in the presence of 20 μM/mL Polybrene. After 72 h, puromycin was added to final concentration of 2 μg/mL for selection. GFP immunofluorescence was assessed by using an Olympus IX71 microscope (Olympus, Tokyo, Japan) filtered with 560-nm excitation and 645-nm emission filters. The cells were grown to 80% confluence in the presence of 2 μg/mL puromycin prior to Western blot.

### 3.8. Real-Time PCR

Total RNA from cells was extracted using Trizol reagent and cDNA was made with a Superscript II reverse transcription kit (Invitrogen, Inc., Carlsbad, CA, USA). The primers for real-time PCR were as followings: for HIF-1α: 5′-GAAGACATCGCGGGGAC-3′ (sense) and 5′-GAAGACATCGCGGGGAC-3′ (antisense), and for GAPDH (glyceraldehyde-3-phosphate dehydrogenase): 5′-CTCCTCTGACTTCAAC AGCG-3′ (sense) and 5′-GCCAAATTCGTTGTCATACCAG-3′ (antisense). The cDNA was amplified by using 10 μL of Master Mix from the DyNAmo SYBR green quantitative real-time PCR kit (MJ Research, Inc., Waltham, MA, USA), 1 mM of each primer, and 2 μL of the cDNA product in a 20-μL total volume. Thirty cycles of 1 min at 94 °C, 30 s at 55 °C, and 40 s at 72 °C were followed by 10 min at 72 °C in an MJ Research Opticon II thermocycler (MJ Research, Inc., Waltham, MA, USA). A melting curve analysis was performed to verify the specificities of the amplified products. The values for the relative levels of change were calculated by the “delta delta threshold cycle” (ΔΔ*C*_t_) method and each sample was tested in triplicates.

### 3.9. Statistical Analysis

Statistical analysis was carried out using Microsoft Excel software to assess differences between experimental groups. Statistical significance was analyzed by Student’s *t* test and expressed as a *p* value. *p* values lower than 0.05 were considered to be statistical significance. Three asterisks indicate *p* < 0.01.

## 4. Conclusions

In summary, we propose a model depicting an important role of HPV16 E6 in the regulation of hypoxia-enhanced glycolysis. Under normoxic conditions, HIF-1a is subjected to VHL-mediated ubiquitination and subsequent rapid degradation. Under hypoxic conditions or within solid tumor microenvironments, however, HPV16 E6 may disrupt the HIF-1a-VHL interaction, stabilizes HIF-1a, and increases expression of HIF-1a-responsive genes, thereafter contributing Warburg effect.
